# Efficacy and Safety of Levetiracetam vs. Phenobarbital for Neonatal Seizures: A Systematic Review and Meta-Analysis

**DOI:** 10.3389/fneur.2021.747745

**Published:** 2021-11-18

**Authors:** Meng-Yuan Qiao, Hong-Tao Cui, Ling-Zhi Zhao, Jing-Kun Miao, Qi-Xiong Chen

**Affiliations:** ^1^Chongqing Traditional Chinese Medicine Hospital, Chongqing, China; ^2^Children's Hospital of Chongqing Medical University, Chongqing, China; ^3^Chongqing Health Center for Women and Children, Chongqing, China

**Keywords:** levetiracetam, phenobarbital, neonatal seizure, meta-analysis, efficacy

## Abstract

**Background:** Neonatal seizures are a common neurological emergency in newborns. Phenobarbital (PB) is the first-line antiepileptic drug (AED). However, PB has some side effects, such as hypotension and respiratory depression, and it can accelerate neuronal apoptosis in the immature brain. Levetiracetam (LEV), a new antiepileptic drug, has been used as a second-line drug for the treatment of neonatal seizures. Compared with PB, LEV has many advantages, including a low incidence of side effects and better neurodevelopmental outcomes. However, there are only a few systematic reviews of LEV for the treatment of neonatal seizures.

**Objective:** To evaluate the efficacy and safety of LEV for neonatal seizures and to compare the efficacy, side effects, and neurological outcomes between LEV and PB in the treatment of neonatal seizures.

**Methods:** The keywords LEV, PB, and neonatal seizure were searched in the MEDLINE, Cochrane Library, Web of Science, EMBASE, clinicaltrials.gov, and China National Knowledge Internet (CNKI) databases with a last update in July 2021 to collect high-quality studies. We collected studies studying the efficacy or safety of LEV and PB in the treatment of neonatal seizures applying strict inclusion and exclusion criteria. The data were extracted and outcome measures, including efficacy, side effect rate, neurological score, and mortality rate, were analyzed with RevMan 5.3 software.

**Results:** Ten articles were finally included in the meta-analysis. The meta-analysis showed that there was no difference in efficacy between LEV and PB in the treatment of neonatal seizures. Compared with PB, the incidence of side effects of LEV was lower. The incidence of hypotension and respiratory depression in the LEV group was significantly lower than that in the PB group. In terms of long-term neurodevelopmental outcomes, there was no significant difference in the Bayley Scales of Infant Development (BSID) scores between LEV and PB.

**Conclusion:** PB is still the first-line AED recommended by the WHO for the treatment of neonatal seizures. The new AEDs LEV may not have better efficacy than PB. At the same time, LEV is associated with better neurodevelopment outcomes and a lower risk of adverse effects. In addition, continuous EEG monitoring should be used to diagnose neonatal seizures to evaluate the severity of the seizures, remission, and drug efficacy.

**Systematic Review Registration:** PROSPERO, identifier: CRD42021279029.

## Introduction

Neonatal seizures are one of the common neurological emergencies in newborns. The incidence of neonatal seizures in full-term infants is 1–3.5‰, in premature infants of 1,500–2,500 g it is 4.4–13.5‰, and in premature infants with birth weight < 1,500 g it is 57.5–1,132‰ (1). Compared with other age groups, the incidence of seizures is higher in the neonatal period. Although most infants with seizures have a good prognosis, 20–30% have seizures that are difficult to control, and most of these have a poor prognosis and long-term neurological sequelae, including cerebral palsy, intellectual disability, and epilepsy. Phenobarbital (PB) is still the first-line antiepileptic drug (AED). It can not only control seizures but also reduce the metabolism of the brain (2). PB can control 43–80% of electrical seizures (abnormal electroencephalograms) in newborns (3, 4). Some children need to use other AEDs as second-line or third-line treatments. However, PB has some side effects, such as hypotension and respiratory depression, and it can lead to cognitive decline in infants and young children (5, 6). At the same time, some studies have found that PB can accelerate neuronal apoptosis in the immature brain.

Levetiracetam (LEV) is a new AED. It was approved by the FDA for clinical antiepileptic treatment in 2012. At present, LEV has been used as a second-line drug for the treatment of neonatal seizures, and the seizure control rate is 35–86% (7, 8). Studies have confirmed that LEV has a neuroprotective effect and does not cause neuronal apoptosis or disrupt synaptic development (9). The Hammersmith neurological examination (HNNE) score in newborns treated with LEV was better than that in the PB group. At the same time, the use of LEV had a significant positive effect on the tone and posture development of the infants (5). Compared with LEV, the neurological prognosis of newborns in the PB group was worse (10). In the past decade, because of the good efficacy, high safety, and good pharmacokinetic characteristics of LEV, it has been increasingly widely used in the treatment of seizures (including neonatal seizures) (11).

LEV is a pyrrolidine derivative that regulates the release of neurotransmitters in synaptic vesicles by binding to synaptic vesicle protein 2A (SV2A) to control seizures (12). Compared with PB, LEV has many advantages, including a lower incidence of side effects and better neurodevelopmental outcomes (3, 5, 10). Furthermore, unlike PB, LEV does not appear to promote neuronal apoptosis in animal models (13) and may have neuroprotective and antiepileptogenic effects (14, 15). For infantile epilepsy, LEV may be more effective than PB for initial monotherapy of non-syndromic epilepsy (16). At the same time, LEV is associated with a lower risk of major malformations than PB during pregnancy (17). After neonatal exposure to PB and LEV, fewer cognitive and motor impairments were seen at 24 months in the LEV group than in the PB group (10). Therefore, LEV may replace PB as the first-line drug for the treatment of neonatal seizures in the future. To date, there are only a few systematic reviews of LEV for the treatment of neonatal seizures. A recent review included only 4 retrospective and 1 prospective study (18). A study directly evaluated the efficacy of LEV vs. PB, but the quality of evidence was very low. With the wide application of LEV, many high-quality studies have been published in recent years. In this study, we systematically evaluated the existing evidence of LEV for the treatment of neonatal seizures and performed a meta-analysis to compare the efficacy and safety of LEV and PB in the treatment of neonatal seizures.

## Methods

### Search Strategy

We searched for studies on the treatment of neonatal seizures with PB and LEV in the Medline, Cochrane Library, Web of Science, EMBASE, clinicaltrials.gov, and China National Knowledge Internet (CNKI) databases with a last update in July 2021 using the keywords LEV, PB, and neonatal seizure. Search words included infant or newborn or neonat^*^, seizure^*^ or epileps^*^ or convulsi^*^, and anticonvuls^*^ or antiepileptic^*^. The types of studies included randomized controlled trials, cohort studies, and case-control studies. The search terms and limits are provided in the supporting information (Tables e-1–e-6).

### Inclusion and Exclusion Criteria

#### Inclusion Criteria

① All of the subjects were neonatal seizure patients [including seizures diagnosed by clinical or electroencephalogram (EEG)]. There was no limitation on gender, race, or other basic characteristics.
② Interventions: Neonatal seizures treated with PB or LEV as the first-line treatment.
③ Outcome measures: The study reported at least one outcome measure considered in our study, such as effectiveness, safety, and neurological prognosis.

#### Exclusion Criteria

① Neonatal seizures caused by electrolyte disorders (such as hypoglycemia and correctable hypocalcemia), metabolic disorders (such as non-ketotic hyperglycemia and pyridoxine deficiency), or opioid withdrawal.
② Case reports, review articles without original data, and articles with incomplete or non-standardized data (e.g., the article does not include the outcomes or outcome data required for analysis in this meta-analysis) were excluded.
③ Studies that included seizures at ages other than neonates were excluded.
④ Studies with a total sample size of fewer than 10 cases were excluded.

### Outcome Measures

#### Efficacy Outcome Measures

After LEV or PB monotherapy, the seizure stopped for at least 24 h or longer (48 h−7 d). Cessation of seizures is defined as the disappearance of clinical seizures. Seizure arrest is defined as the disappearance of clinical seizures (e.g., no abnormal gaze or eye movement, tongue extension, apnea, clonus, tonic or convulsive movements, etc.) and/or normal EEG monitoring. At the same time, we also included a reduction in seizures by more than 50%.

#### Adverse Effects

Adverse effects included hypotension, respiratory depression, heart rate abnormalities, poor feeding, irritability, infection, and anorexia. Considering that hypotension and respiratory depression were the most common drug side effects, this study performed subgroup analysis on the occurrence of different side effects.

#### Neurological Prognosis

Neurological development was followed up and a poor prognosis was defined as intellectual disability, cerebral palsy, epilepsy, and other complications, and the neurobehavioral score (Bayley Scales of Infant development-III, BSID-III) was analyzed.

### Data Extraction and Literature Evaluation

#### Data Extraction

A specification data extraction form was predesigned, and two authors (Q.M.Y. and C.H.T.) independently extracted data using the form. Discrepancies were resolved through discussion with other authors (Z.L.Z. and C.Q.X.). The extracted data included:

① Basic information: title, year, journal, impact factor (IF), first author, country, type of publication, etc.
② The qualifications of the included study: whether the participants were neonates with definite seizures, whether the intervention measures met the requirements, etc.
③ Characteristics of the subjects: sample size, grouping sample size, gestational age, sex, birth weight, etc.
④ Intervention: the total dose and course of treatment of PB and LEV.
⑤ Outcomes: efficacy, safety, and neurological system.
⑥ Elements of risk assessment of bias in different study types.

#### Literature Evaluation

Two authors (Q.M.Y. and C.H.T.) independently evaluated the quality of the study. Discrepancies were resolved through discussion with the other authors (Z.L.Z. and C.Q.X.). We adopted different evaluation methods according to the different types of study research. The Cochrane risk of bias tool was used to evaluate the quality of the randomized controlled trials (RCTs). It includes seven items: random sequence generation, allocation concealment, blinding of participants and personnel, blinding of outcome assessment, incomplete outcome data, selective reporting, and other bias. Each item was divided into low-risk, unknown, and high-risk (19). Cohort studies and case-control studies were evaluated by the Newcastle-Ottawa Scale (NOS): there were 8 items in 3 sections with 9 scores, including the selection of the study population, comparability between groups, and the measurement of exposure factors, among which ≥6 was a high-quality study (20).

### Statistical Analysis

We performed a meta-analysis if data were available using RevMen5.3, and the odds ratio (OR) and 95% CI were calculated. The included studies were tested for heterogeneity by the chi-square test. According to the system evaluation manual, the significance level of heterogeneity was *P* = 0.1, *I*^2^ = 50%:

① The fixed-effects model was used for analysis if *P* > 0.1 and *I*^2^ ≤ 50%, which meant that the studies had good homogeneity and heterogeneity.
② The random-effects model was used for analysis if *P* ≤ 0.1 and *I*^2^ > 50%, which meant that there was significant heterogeneity between studies. Subgroup analysis or sensitivity analysis was carried out to identify the sources of heterogeneity. Finally, a funnel plot was used to evaluate the publication bias.

## Results

### Search Strategy

A total of 12,434 relevant articles were initially retrieved (see Table 1). We read the full text of 320 studies and finally included 10 studies with a total of 930 patients.

**Table 1:** The flow chart of literature search and screen.

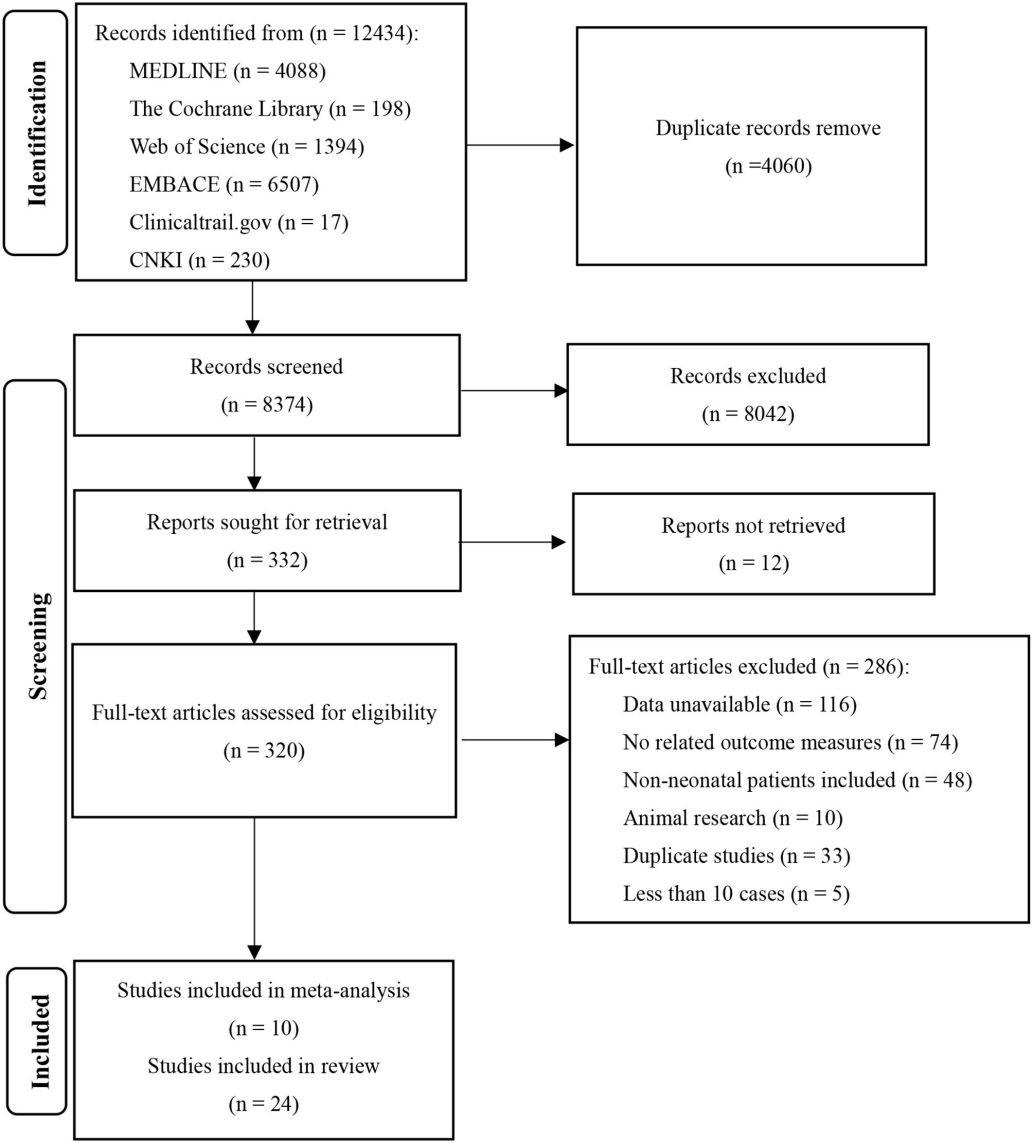

### Characteristics of the Included Studies

Ten studies were included in this study (3, 5, 10, 21–27), including 8 in English and 2 in Chinese. A total of 930 participants were included, and individual study sample sizes ranged from 22 to 280 patients. Four articles were retrospective studies, 2 were prospective cohort studies, and 4 were randomized controlled trials. Three studies used clinical seizures as the diagnostic criteria, 4 studies used EEG abnormalities as the diagnostic criteria, and 3 studies included neonatal seizures diagnosed by clinical or EEG seizures. The doses of PB were mostly between 10 and 20 mg/kg/d. The dose of LEV was between 20 and 60 mg/kg/d. As detailed in Table 2, 6 studies reported efficacy outcomes and 5 studies reported drug-related side effects. As detailed in Table 3, 4 studies reported neurological outcomes.

**Table 2:** Characteristic of included studies on efficacy analysis (6 studies).

**References**	**Study type**	**Diagnostic criteria**	**Intervention group**		**Efficacy and numbers (%)**	**Side effects (%)**	**Death (%)**
			**PB**	**LEV**			
Li et al. (24)	RCT	v-EEG confirmed seizures	*N* = 31 GA, (wk): 39.1 Male, n (%):17 (57%) BW, kg [median (IQR)]: 3.4 (2.5–5.0) Dose: 10–20 mg/kg/d iv	*N* = 30 GA, (wk): 39.4 Male, *n* (%):18 (48%) BW, kg [median (IQR)]:3.4 (2.5–4.8) Dose: 30–60 mg/kg/d po	PB: SF 24 h 8/31 (26%), 2–7 d 9/31 (30%), Total 17/31 (55%) LEV: SF 24 h 16/30 (53%), 2–7 d 4/30 (13%), Total 20/30 (67%)	PB: Urinary Retention 1/31 (3%) LEV: 0/3	ND
Tan et al. (23)	Prospective study	Clinical confirmed seizures	*N* = 35 GA, (wk): ND Male, *n* (%):21 (57%) BW, kg [median (IQR)]: 4.6 (3.0–6.7) Dose: 10–20 mg/kg/d iv	*N* = 35 GA, (wk): ND Male, *n* (%):19 (48%) BW, kg [median (IQR)]: 4.7 (3.1–6.8) Dose: 30–60 mg/kg/d po	PB: SR 10 h 29/35 (83%) LEV: SR 10 h 34/35 (97%)	PB: Increased respiratory secretions 7/35 (20%), Respiratory depression 3/35 (9%), Rash 1/35 (3%) LEV: Increased respiratory secretions 1/35(3%), Respiratory depression 2/35 (6%)	ND
Gowda et al. (25)	RCT	Clinical confirmed seizures	*N* = 50 GA: ND Male, *n* (%):28 (56%) BW, kg: 2.73 Dose: 20–30 mg/kg/d	*N* = 50 GA: ND Male, *n* (%):28 (56%) BW, kg: 2.56 Dose: 20–40 mg/kg/d	PB: SF 24 h 31/50 (62%) LEV: SF 24 h 43/50 (86%)	PB: Hypotension 5/50 (10%), Bradycardia 3/50 (6%), Mechanical ventilation is required 2/50 (4%) LEV: 0/50	ND
Thibault et al. (21)	Retrospective single center study	c-EEG confirmed seizures	*N* = 31 GA, (wk) [median (IQR)]: 38 (37–39) Male, *n* (%): 20 (35.5%) BW, kg [median (IQR)]: 3.2 (2.9–3.5) Dose: 10–20 mg/kg/d	*N* = 22 GA, (wk) [median (IQR)]: 38 (37–39) Male, *n* (%): 10 (54.5%) BW, kg [median (IQR)]: 3.1 (2.3–3.5) Dose: 20–30 mg/kg/d	PB: SF 18/31 (58%) LEV: SF 12/22 (55%)	PB: Hypotension 7/31 (23%), Respiratory depression 1/31 (3%) LEV: 0/22	ND
Sharpe et al. (3)	RCT	c-EEG confirmed seizures	*N* = 42 GA, (wk) [median (IQR)]: 39.1 (38.3–40.3) Male, *n* (%):24 (57%) BW, kg [median (IQR)]: 3.3 (2.9–3.7) Dose: 20–40 mg/kg/d	*N* = 64 GA, (wk) [median (IQR)]: 39.3 (38.3–40.3) Male, *n* (%): 31(48%) BW, kg [median (IQR)]: 3.3 (3.0–3.6) Dose: 40–60 mg/kg/d	PB: SF 24 h 24/30 (80%) LEV: SF 24 h 15/53 (28%)	PB: Serious side effects 5/42 (12%) LEV: Serious side effects 4/64 (6%) PB: Hypotension 7/42 (17%), Respiratory depression 11/42 (26%) LEV: Hypotension 3/64 (5%), Respiratory depression 8/64 (12%)	PB: 1/42 (2%) LEV: 2/64 (3%)
Wagner et al. (22)	Retrospective cohort study	Clinical or v-EEG confirmed seizures	*N* = 73 BW, (wk) (M ± SD): 36.01 ± 5.1 Male, *n* (%): ND BW, kg [median (IQR)]: 2.7 (2.0–3.2) Dose: 15.7–23.6 mg/kg/d	*N* = 73 BW, (wk) (M ± SD): 37.91 ± 2.33 Male, *n* (%): ND BW, kg [median (IQR)]: 3.0 (2.5–3.5) Dose: 35.4–60 mg/kg/d	PB: SF after elimination of BZDs 29/56 (52%) LEV: SF after elimination of BZDs SF 26/51 (51%)	ND	ND

*PB, phenobarbital; LEV, levetiracetam; BZDs, benzodiazepines; RCT, Random Clinical Trail; GA, Gestation Age; BW, Birth Weight; SF, Seizure Free; SR, Seizure Reduce; ND, Not Data; v-EEG, Video electroencephalography; c-EEG, Continuous electroencephalography; IQR, interquartile range; M ± SD, mean ± standard deviation*.

**Table 3:** Characteristics of included studies on neurodevelopmental outcomes (4 studies).

**References**	**Study type**	**Diagnostic criteria**	**Number**	**Neurodevelopment**		**CP (%)**	**Death (%)**	**Other AEDs**
Maitre et al. (10)	Retrospective study	Clinical confirmed seizures	LEV = 174 PB = 247	BSID-III ^a^: LEV (24 m)	Cognition: 85 (60–93)	LEV: 28/174 (16%) PB: 67/247 (27%)	LEV: 31/174 (18%) PB: 67/247 (27%)	A total of 141 patients received PB combination with LEV
					Motor: 85 (69–94)			
					Language: 82 (67–95)			
				BSID-III ^a^: PB (24 m)	Cognition: 85 (70–95)			
					Motor: 85 (65–96)			
					Language: 89 (71–97)			
Ghosh et al. (26)	Retrospective cohort study	Clinical or EEG confirmed seizures	LEV = 7 PB = 15	BSID-III ^a^: LEV (9–14 m)	Cognition: 70 (60–83.75)	ND	ND	2 patients received cross treatment
					Motor:61 (49–92.5)			
					Language: 86 (77–97)			
				BSID-III ^a^: PB (9–14 m)	Cognition:85 (65–90)			
					Motor:82 (58–86.5)			
					Language:86 (72.5–94.5)			
Falsaperla et al. (5)	Randomized single blind prospective study	EEG confirmed seizures	LEV = 15 PB = 15	HNNE ^b^: LEV	Before treatment: 27.33 ± 4 03	ND	ND	0
					A month after treatment: 32.4 ± 1.75			
				HNNE ^b^: PB	Before treatment: 27.83 ± 3.25			
					A month after treatment: 28.63 ± 2.73			
Arican et al. (27)	Prospective study	Clinical confirmed seizures	LEV = 40 PB = 22	BSID-III ^b^: LEV (18–24 m)	Cognition: 84.6 ± 28.1	ND	ND	0
					Motor:83.6 ± 34.4			
					Language: 82.6 ± 31.7			
				BSID-III ^b^: PB (18–24 m)	Cognition:90 ± 27.6			
					Motor:94.3 ± 34.8			
					Language:89.1 ± 31.3			

a *Data was presented as median (interquartile range)*.

b *Data was presented as mean ± standard deviation (M ± SD)*.

*PB, phenobarbital; LEV, levetiracetam; BSID-III, Bayley Scales of Infant development-III; ND, Not date; CP, cerebral palsy; HNNE, Hammersmith Neonatal Neurological Examination*.

### Study Quality and Publication Bias

**Figure 1:**
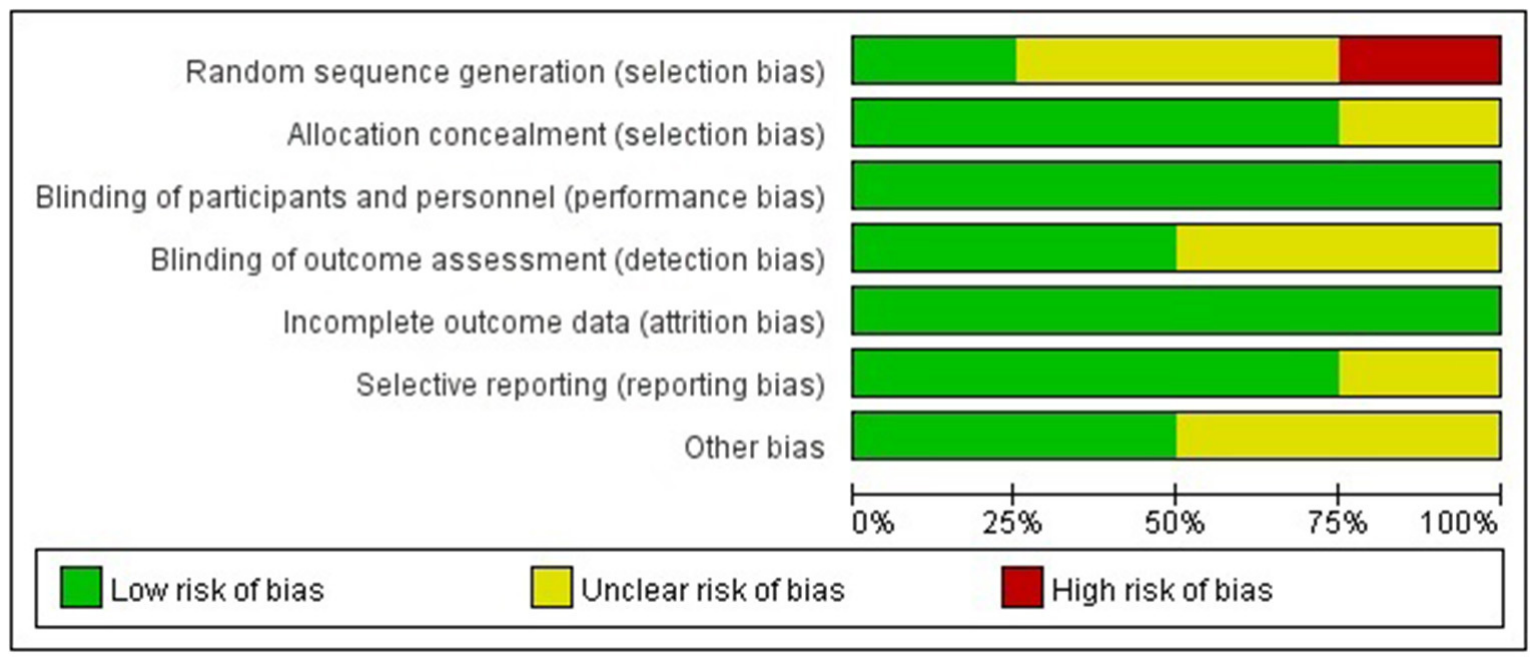
Quality evaluation of the included RCTs.

**Figure 2:**
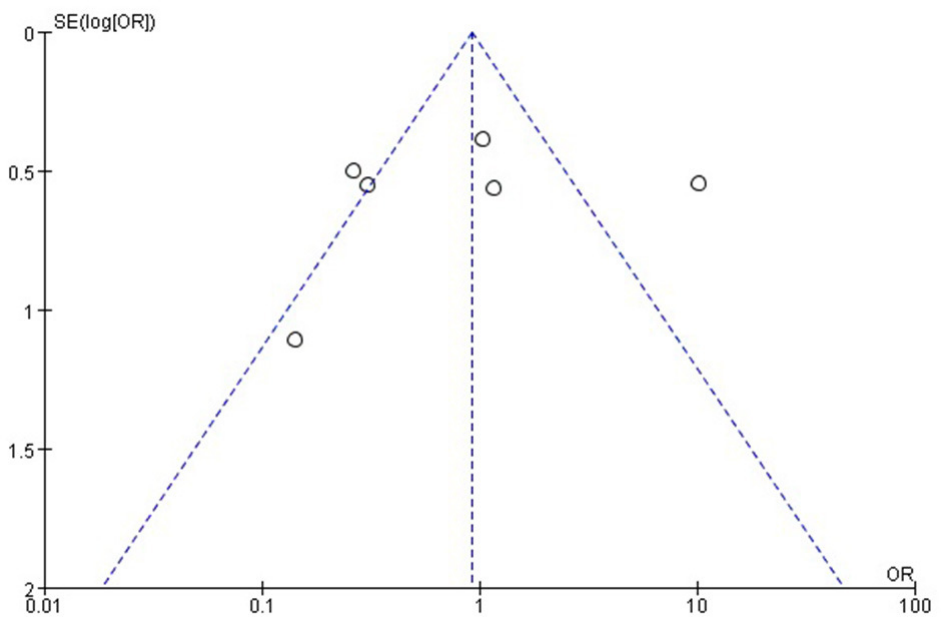
Funnel plot. OR, odds ratio.

The Cochrane risk of bias tool was used to evaluate the quality of the RCTs. As shown in Figure 1, each item was divided into low risk, unclear, and high risk. The NOS was used to evaluate the quality of cohort studies and case-control studies, for which ≥6 was considered a high-quality study (see Table 4). The results suggested that all studies were high quality. Risk of bias was assessed for the 6 articles included in the efficacy analysis. The funnel plot distribution was approximately symmetrical, indicating that there was no publication bias in this field (see Figure 2).

**Table 4:** Quality evaluation of the included cohort studies or case control studies (NOS).

**References**	**Selection**	**Comparability**	**Outcome**	**Overall score**
Maitre et al. (10)	4	0	3	7
Tan et al. (23)	3	1	3	7
Ghosh et al. (26)	4	1	3	8
Arican et al. (27)	3	2	2	7
Thibault et al. (21)	4	2	3	9
Wagner et al. (22)	3	2	3	8

*NOS, Newcastle-Ottawa Scale*.

### Efficacy Analysis

**Figure 3:**
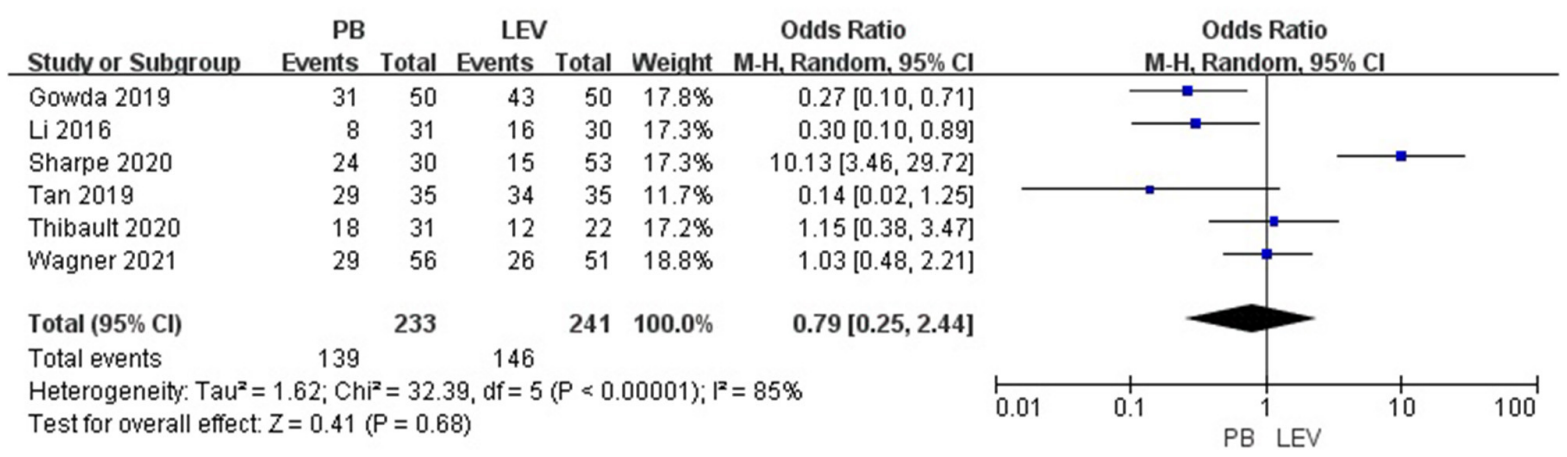
Comparison of efficacy in the PB and LEV groups. M-H, Mantel–Haenszel; CI, confidence interval.

Six studies reported the efficacy of LEV compared with PB for neonatal seizures (3, 21–25). The results are shown in Figure 3. There was no significant difference in efficacy between LEV and PB for treating neonatal seizures [OR = 0.79, 95% CI (0.25–2.44), *P* = 0.68]. Subgroup analysis was performed according to the different study types (see Table 5). In three study types (randomized controlled trials, prospective cohort studies, and retrospective studies), no significant difference in efficacy was observed between LEV and PB (*P* = 0.95, *P* = 0.83, *P* = 0.08), showing that different types of studies had no influence on the efficacy analysis. However, statistical heterogeneity between individual studies was noted using the *I*^2^ statistic (*I*^2^ = 85%). The results of the subgroup analysis showed that the diagnostic criteria of seizures and different doses of PB all led to heterogeneity (see Table 5). Clinical-confirmed seizures were used as the diagnostic criteria in 2 studies (23, 25), and EEG-confirmed seizures were used as diagnostic criteria in 3 studies (3, 21, 24). Subgroup analysis was performed according to the different diagnostic criteria. The results showed that for neonates with clinical seizures, the efficacy of the LEV group was better than that of the PB group [OR = 0.24, 95% CI (0.10–0.58), *P* = 0.002]. However, for neonates with an EEG diagnosis, there was no significant difference in efficacy between the two groups [OR = 1.53, 95% CI (0.20–11.50), *P* = 0.68]. The difference might be caused by the atypical clinical manifestations of neonatal seizures, which lead to the inability to clearly observe the efficacy of drugs. Therefore, we recommend that continuous EEG monitoring should be used to diagnose neonatal seizures to evaluate the severity of the seizures, remission, and drug efficacy. Using different doses of PB also led to heterogeneity. The efficacy of high-dose PB (20–40 mg/kg/d) was better than that of LEV [OR = 10.13, 95% CI (3.46–29.72), *P* < 0.0001], while the efficacy of medium-dose PB (20–30 mg/kg/d) was inferior to that of LEV [OR = 0.27, 95% CI (0.10–0.71), *P* = 0.008]. However, there was only one study each of high-dose (3) and medium-dose PB (25) for the treatment of neonatal seizures. Due to the lack of studies, it is still unclear whether the efficacy of different doses of PB is superior or inferior to LEV.

**Table 5:** Subgroup analysis in efficacy between PB and LEV.

	**Subgroup**	**OR**	**95% CI**	***P***
Study type	Random clinical trails	0.93	0.09–9.41	0.95
	Retrospective study	1.07	0.57–2.00	0.83
	Prospective study	0.14	0.02–1.25	0.08
	Total	0.79	0.25–2.44	0.68
Different diagnostic	Clinical confirmed seizures	0.24	0.10–0.58	0.002
criteria of seizures	EEG confirmed seizures	1.53	0.20–11.50	0.68
	Clinical or EEG confirmed seizures	1.03	0.48–2.21	0.93
	Total	0.79	0.5–2.44	0.68
Different doses	10–20 mg/kg/d	0.61	0.27–1.39	0.24
of PB	20–30 mg/kg/d	0.27	0.10–0.71	0.008
	20–40 mg/kg/d	10.13	3.46–29.72	<0.0001
	Total	0.79	0.5–2.44	0.68

*OR, odds ratio; CI, confidence interval*.

Sensitivity analysis was carried out. The results show that the study of Sharpe et al. had a large influence on the heterogeneity of research. We found that the *I*^2^ decreased to 56% when Sharpe's study was removed (3). However, after removing this study, there was still no significant difference in efficacy between the two groups [OR = 0.50, 95% CI (0.24–1.06), *P* = 0.07], indicating that the result of the meta-analysis was stable.

### Adverse Effects

**Figure 4:**
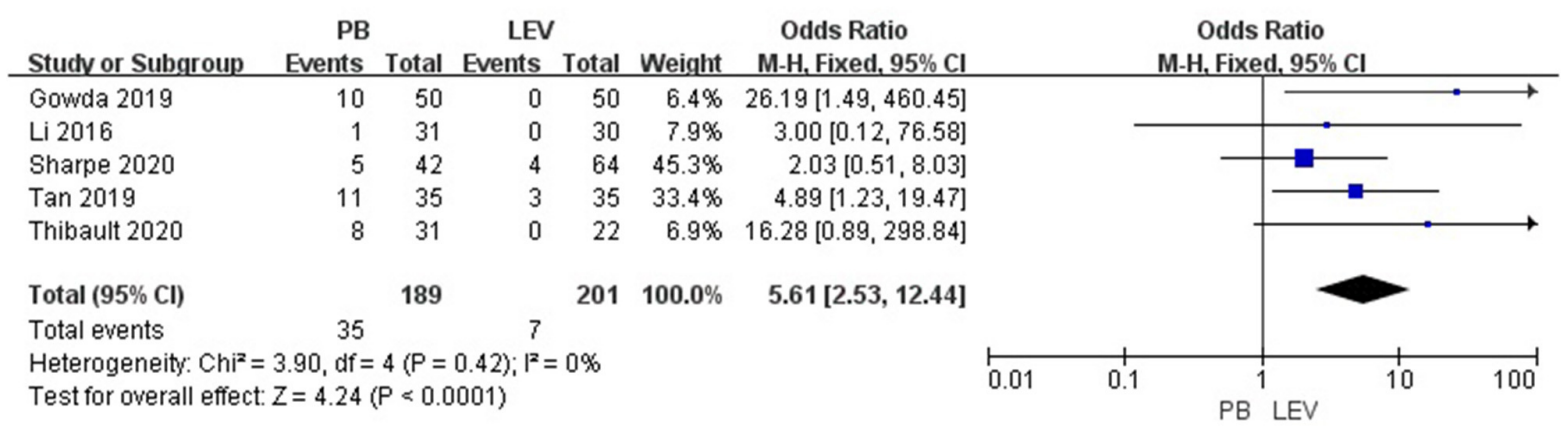
Comparison of adverse effects in the PB and LEV groups. M-H, Mantel–Haenszel; CI, confidence interval.

Five studies reported the adverse effects of LEV and PB for the treatment of neonatal seizures (3, 21, 23–25). The results are shown in Figure 4: PB has a higher incidence of adverse effects in treating neonatal seizure than LEV, which means that LEV has fewer adverse effects, and the difference was statistically significant [OR = 5.61, 95% CI (2.53–12.44), *P* < 0.0001]. Hypotension and respiratory depression were the most common adverse effects. Three studies reported medication-related hypotension (3, 21, 25). The incidence of hypotension in the LEV group was 0–5%, while that in the PB group was as high as 0–23%. Four studies reported the occurrence of respiratory depression during treatment (3, 21, 23, 25). The incidence of respiratory depression in the LEV group was 0–12%, while that in the PB group was as high as 0–26%. Subgroup analysis was carried out on the adverse effects (see Table 6). Significant differences were found for the incidence of hypotension and respiratory depression, which were higher in the PB group than in the LEV group [OR = 6.84, 95% CI (2.18–21.44), *P* = 0.001; OR = 2.41, 95% CI (1.06–5.46), *P* = 0.04]. However, no statistically significant difference was found in the incidence of bradycardia or increase in respiratory secretions between the two groups (*P* = 0.19, *P* = 0.05).

**Table 6:** Subgroup analysis in adverse effects between PB and LEV.

**Subgroup**	**OR**	**95% CI**	***P***
Hypotension	6.84	2.18–21.44	0.001
Respiratory depression	2.41	1.06–5.46	0.04
Bradycardia	7.44	0.37–147.92	0.19
Increased respiratory secretions	8.50	0.99–73.28	0.05

*OR, odds ratio; CI, confidence interval*.

### Neurodevelopmental Outcomes

Three articles (10, 26, 27) reported the BSID scores of neonatal seizures treated with LEV or PB, and they reported scores for various domains (cognitive, motor, language). The results are shown in Table 7. There was no difference in cognitive and motor scores between the LEV and PB groups [SMD = 0.05, 95% CI (−0.13 to 0.23), *P* = 0.57; SMD = 0.07, 95% CI (−0.11 to 0.24), *P* = 0.47]. However, the language score in the PB group was higher than that in the LEV group [SMD = 0.32, 95% CI (0.14–0.50), *P* = 0.0005]. This is not consistent with previous studies. After removing Maitre's study during a sensitivity analysis (10), there was no difference in language scores between the two groups. In the study of Maitre, 141 people were treated with PB in combination with LEV, which may have impacted the BSID score.

**Table 7:** Comparison of BSID scores in the PB and LEV groups.

**BSID**	**SMD**	**95% CI**	***P***
Cognitive	0.05	−0.13 to 0.23	0.57
Motor	0.07	−0.11 to 0.24	0.47
Language	0.32	0.14–0.50	0.0005

*BSID, Bayley Scales of Infant development; SMD, standardized mean difference; CI, confidence interval*.

## Discussion

### Summary of the Main Results

At present, AEDs are still the main treatment for neonatal seizures. PB is still the first-line treatment recommended by the WHO because it can not only control seizures but also reduce the brain metabolic rate. PB has been proven to be able to control neonatal seizures caused by various etiologies. Existing studies have shown that the efficacy of PB in controlling neonatal seizures is 43–80% (3, 4). Some children with refractory seizures need to be treated with second-line or third-line AEDs. However, there are still many problems because of the adverse effects of PB, such as hypotension, respiratory depression, abnormal heart rate, poor feeding, and hypothermia (6, 28). At the same time, some studies have reported that PB may damage the developing brain, accelerate nerve cell apoptosis, and cause cognitive impairment. Therefore, its safety and neurodevelopmental prognosis need to be evaluated. LEV, a new type of AED, has many advantages including a low incidence of side effects and little neurological harm. Randomized controlled trials and observational studies have shown that the incidence of serious side effects such as hypotension and mechanical ventilation under treatment with LEV is lower than that under treatment with PB (18). Thus, LEV has been increasingly used for the treatment of neonatal seizures, and it may replace PB as the first-line drug for the treatment of neonatal seizures. In recent years, there have been many high-quality studies comparing the efficacy of LEV and PB for the treatment of neonatal seizures. Therefore, we evaluated the efficacy and safety of LEV for neonatal seizures and performed a meta-analysis to evaluate the efficacy and safety of LEV vs. PB for neonatal seizures.

We evaluated 24 studies (see Table 8) on LEV for the treatment of neonatal seizures, including 15 retrospective studies (7, 8, 21, 22, 31, 32, 35, 37–44), 6 prospective studies (29, 30, 33, 34, 36), and 3 RCTs (3, 24, 25). In these studies, the dosage of LEV was mostly between 10 and 60 mg/kg/d. Thirteen studies used electrical seizures as the diagnostic criteria for newborns (3, 7, 8, 21, 24, 30–32, 34, 37, 38, 41, 43), 5 studies used clinical or electrical seizures as the diagnostic criteria (22, 29, 35, 39, 40), and 6 studies confirmed seizures only by clinical manifestations (23, 25, 33, 36, 42, 44). Nine studies defined seizure cessation on EEG within 24 h as a primary outcome (3, 8, 24, 25, 31, 32, 34, 37, 39), some studies contained longer seizure control times (48 h−7 d) (29, 30, 33, 38, 44), and 7 articles did not describe the seizure control times (21, 22, 35, 36, 40, 41, 43). One study defined seizure remission ≥ 50% by EEG within 24 h as the primary outcome (7), and another study defined seizure reduction ≥ 50% clinically as the secondary outcome (42). In most studies, LEV was used as the second-line or third-line drug. Most of the studies, including one in particular (41), considered LEV to have good efficacy (35–100%). In most studies, more than 50% of neonates stopped their seizures after LEV treatment. When LEV was used as a second-line or third-line drug for the treatment of neonatal seizures, the seizure control rate was 35–100%. When LEV was used as the first-line treatment, the seizure control rate within 24 h was 28–86%, and the seizure control rate within 7 d was 79–100%. When LEV was combined with PB or PHT for the treatment of neonatal seizures, the effective rate was as high as 83% (40). The incidence of adverse effects of LEV is 0–12% (3, 23, 42), and the most common adverse effects are respiratory depression and hypotension, which occur in 0–12% and 0–5% of cases, respectively. In a retrospective study (43), high-dose LEV had no adverse effects. In 5 prospective studies, only one study (23) reported adverse effects of LEV: one patient had respiratory secretions and 3 patients had respiratory inhibition. Therefore, it is concluded that LEV has a low incidence of side effects and high safety.

**Table 8:** Studies on levetiracetam for the treatment of neonatal seizures (24 studies).

**References**	**Type**	**Diagnostic Criteria**	**N**	**The doses of LEV (mg/kg/day)**	**Efficacy and Numbers (%)**	**Adverse effects (%)**	**Neurodevelopment**	**Other AEDs**
Fürwentsches et al. (29)	Prospective cohort study	Clinical confirmed seizures	6	po 10–50 mg/kg/d	SR 6 d (100%)	0	ND	5 patients received PB before LEV and 5 patients received PB after PB
Abend et al. (7)	Retrospective study	c-EEG confirmed seizures	23	10–64 mg/kg/d	SR≥50% 24 h 8 (35%) SR≥50% 24–72 h 12(52%)	0	ND	5 patients received PB and 2 received PB + PHT before LEV
Khan et al. (8)	Retrospective study	EEG confirmed seizures	22	10–50 mg/kg/d	SF 24 h 14 (64%), 48 h 19 (86%), 72 h 22 (100%)	0	ND	16 patients received other ADEs before LEV
Ramantani et al. (30)	Prospective study	EEG confirmed seizures	38	10–65 mg/kg/d	SF 7 d 30 (79%)	0	Postnatal epilepsy at 12 months: 17%, developmental delay: 25%	
Khan et al. (31)	Retrospective study	c-EEG confirmed seizures	12	25–50 mg/kg/d	SF 24 h 9(82%), 48–72 h 10(91%)	0	ND	9 patients received PB before LEV
Yau et al. (32)	Retrospective study	EEG confirmed seizures	12	5–60 mg/kg/d	SR 24 h 7 (58%), 72 h 9 (75%)	0	ND	12 patients received PB before LEV
Sedighi et al. (33)	Prospective cohort study	Clinical confirmed seizure	50	20–40 mg/kg/d	SF 7 d 47(94%)	0	ND	0
Li J et al. (24)	RCT	v-EEG confirmed seizures	30	po 30–60 mg/kg/d	SF 24 h 16(53%), 2–7d 4 (13%), Total 20 (67%)	0	ND	10 patients received PB before LEV
Falsaperla et al. (34)	Prospective cohort study	v-EEG confirmed seizures	16	10–64 mg/kg/d	SR 24 h 6 (38%), 48 h 10 (63%), 6 d 14 (88%), 15 d 16 (100%)	0	ND	0
Venkatesan et al. (35)	Retrospective study	Clinical or EEG confirmed seizures	32	20–65 mg/kg/d	SF 27 (84%)	0	ND	32 patients received PB before LEV
Mollamohammadi et al. (36)	Prospective cohort study	Clinical confirmed seizures	42	po 10–50 mg/kg/d	SF 40 (95.3%)	0	ND	42 patients received PB before LEV
Rao et al. (37)	Retrospective study	c-EEG confirmed seizures	18	20–60 mg/kg/d	SF 24 h 9(50%)	0	ND	10 patients received PB before LEV
Han et al. (38)	Retrospective study	c-EEG confirmed seizures	37	20–60 mg/kg/d	SF 48 h 21(57%)	0	ND	0
Tan et al. (23)	Prospective study	Clinical confirmed seizures	LEV = 35 PB = 35	LEV: po 30–60 mg/kg/d PB: iv 10–20 mg/kg/d	LEV: SR 10 h 34/35(97%) PB: SR 10 h 29(83%)	LEV: Increased respiratory secretions 1 (3%), Respiratory depression 2 (6%)	ND	0
Özalkaya et al. (39)	Retrospective study	Clinical or a-EEG confirmed seizures	26	7.7–26.2 mg/kg/d	SF 24 h 16(61%)	0	ND	13 patients received other ADEs before LEV
Kreimer et al. (40)	Retrospective study	Clinical or c-EEG confirmed seizures	36	17.8–61.2 mg/kg/d	SF 17(47%)	0	ND	1 patients received BZDs before LEV
Kurtom et al. (41)	Retrospective study	c-EEG confirmed seizures	61	40–80 mg/kg/d	SF 16(26%)	0	ND	45 patients received PB, fos-PHT after LEV
Gowda et al. (25)	RCT	Clinical confirmed seizures	LEV = 50 PB = 50	LEV: 20–40 mg/kg/d PB: 20–30 mg/kg/d	PB: SF 24 h 31/50 (62%) LEV: SF 24 h 43/50 (86%)	LEV: 0	ND	0
Liu et al. (42)	Retrospective study	Clinical confirmed seizures	LEV = 59 PHB = 66	LEV: po 8–54 mg/kg/d PB: iv 5 mg/kg/d; po 3–11 mg/kg/d	LEV: SF 3 d 12(20%), SR ≥ 50% 16 w 47 (80%) PB: SF 3 d 28(42%), SR ≥ 50% 16 w 38(58%)	LEV: Irritability 3 (6.38%) and anorexia 3 (6.38%).	LEV: 16 w 66.0–76.6% PB: 16 w 50.0–60.5%	0
Thibault et al. (21)	Retrospective study	c-EEG confirmed seizures	LEV = 22 PB = 31	LEV: 20–30 mg/kg/d PB: 10–20 mg/kg/d	LEV: SF 12 (55%) PB: SF 18 (58%)	0	ND	0
Hnaini et al. (43)	Retrospective study	c-EEG confirmed seizures	15	Low dose: 40–60 mg/kg/d High dose: 80–100 mg/kg/d	Low dose SF 6/10 (60%) High dose SF 8/10 (80%)	0	ND	6 patients received PB\PHT\OXC after LEV
Kanmaz et al. (44)	Retrospective study	Clinical confirmed seizures	67	10–50 mg/kg/d	SF 7 d 43(64%)	0	ADSI: Good: 23 (69.7%)	24 patients received combination therapy after LEV
Sharpe et al. (3)	RCT	c-EEG confirmed seizures	LEV = 64 PB = 42	LEV: 40–60 mg/kg/d PB: 20–40 mg/kg/d	PB: SF 24 h 24/30 (80%) LEV: SF 24 h 15/53 (28%)	LEV: Hypotension 3 (5%), Respiratory depression 8 (12%)	ND	0
Wagner et al. (22)	Retrospective study	Clinical or c-EEG confirmed seizures	LEV = 73 PHB = 73	LEV: 35.4–60 mg/kg/d PB: 15.7–23.6 mg/kg/d	LEV: SF 30 (41%) LEV: Seizure control rate after excluding pre-use of BZD drugs 26/51 (51%) PB: SF 45 (62%) PB Seizure control rate after excluding pre-use of BZD drugs 29/56 (52%)	0	ND	22 patients received BDZ before LEV 17 patients received BZD before PB

*PB, phenobarbital; LEV, levetiracetam; PHT, phenytoin; fos-PHT, fos-phenytoin; OXC, Oxcarbazepine; BZDs, benzodiazepines; RCT, Random Clinical Trail; SF, Seizure Free; SR, Seizure Reduce; ND, Not Data; v-EEG, Video electroencephalography; c-EEG, Continuous electroencephalography; IV, intravenous; PO, oral; ADSI, Ankara Developmental Screening Inventory*.

An open-label randomized controlled trial (25) enrolled 100 neonates with clinical seizures. The seizure control rate was 86% (43/50) in the LEV group and 62% (31/50) in the PB group (*P* < 0.01). A prospective study (23) also compared the efficacy of LEV with PB for the treatment of clinically confirmed neonatal seizures and found that the seizure control rate in the LEV group (97.1%) was superior to that in the PB group (82.9%, *P* < 0.01). However, in both studies, neonatal seizures were diagnosed by clinical manifestations, and EEG monitoring and confirmation were not performed. Due to the atypical nature of neonatal seizures, continuous video-EEG monitoring (electrical seizures) has been used as the diagnostic criterion for seizures in recent years. No study has shown that the efficacy of LEV is better than that of PB when using electrical seizures as the diagnostic criterion for seizures. A retrospective cohort study (22) compared the efficacy of LEV and PB for the treatment of neonatal seizures diagnosed by clinical or EEG diagnosis. In that study, for children with seizures who received BZD treatment in advance, the seizure control rate in the PB group was 61.6%, and the seizure control rate in the LEV group was 41.1%. However, in children with seizure who did not receive treatment with BZDs prior, the seizure control rate of LEV group was like that of the PB group (52, 51%), which was like the results of another retrospective study. A retrospective single-center study (21) compared the efficacy of LEV to PB for treating neonatal seizures diagnosed by EEG after cardiopulmonary bypass cardiac surgery, and found that there was no significant difference between LEV and PB as the first-line treatment (54.5%, 58.1%, *P* = 1.0). A multicenter, randomized, double-blind, controlled IIb trial (3) enrolled children diagnosed with electrical seizures. The efficacy of the PB group was better than that of the LEV group (*P* < 0.001), considering PB was a better ADE. After considering all studies' diagnostic criteria, we performed a meta-analysis, and the results showed that there was no significant difference in efficacy between the LEV group and PB group (*P* = 0.68). However, there was heterogeneity in the literature. Subgroup analysis showed that the diagnostic criteria of seizures and different doses of PB were the main causes of the heterogeneity. For the children with clinical seizures, the efficacy of the LEV group was better than that of the PB group (*P* < 0.002). However, there was no significant difference between the LEV group and the PB group (*P* > 0.05) among the children diagnosed by electrical seizures. This is the same as the results of previous studies. Therefore, it is suggested that continuous EEG monitoring should be used to diagnose neonatal seizures to evaluate the severity of seizures, remission, drug efficacy, and so on.

In a retrospective study (21), there were 7 patients with hypotension and 1 patient with respiratory depression in the PB group but no adverse effects in the LEV group (*P* = 0.006). In a randomized controlled trial (25), there were no adverse effects in the LEV group, while the incidences of hypotension, bradycardia, and respiratory depression in the PB group were 10, 6, and 4%, respectively. In the other two studies (3, 23), the incidence of adverse effects in the PB group was higher than that in the LEV group. In our study, meta-analysis showed that the incidence of side effects of LEV was lower than that of PB (*P* < 0.0001). Subgroup analysis showed that the incidence of hypotension and respiratory depression in the LEV group was significantly lower than that in the PB group (*P* = 0.001), which is consistent with previous research results. At the same time, we found that hypotension and respiratory depression were the most common side effects of the two drugs, and the incidences in the PB group were 0–23% and 0–26%, respectively, and those in the LEV group were 0–5% and 0–12%, respectively.

A study (10) found that the long-term neurodevelopmental outcomes (BSID score) of newborns in the LEV group at 24 months of age were better than those in the PB group. A randomized double-blind prospective study (5) found that the HNNE score of newborns treated with LEV was better than that of newborns treated with PB, and the use of LEV had a significant positive effect on tone and posture development (*P* = 0.001). However, in a retrospective cohort study (26), there was no significant difference in BSID scores between the LEV group and the PB group at the age of 9–14 months. Similarly, another study (27) found that there was no difference in BSID scores between the LEV group and the PB group at the age of 18–24 months. We performed a meta-analysis of continuous variables according to the BSID scores. The results showed that there was no significant difference in cognitive and motor scores between the LEV group and the PB group (*P* = 0.57, *P* = 0.47). However, the language score of the PB group was higher than that of the LEV group (*P* = 0.0005). This is not consistent with previous studies. After removing Maitre's study (10) in sensitivity analysis, there was no difference in language scores between the two groups. In the study of Maitre, 141 people were treated with PB in combination with LEV, which may have impacted the BSID score.

### Limitations

This study also has some limitations. First, due to the lack of sufficient randomized clinical trials, observational studies were included, resulting in a decline in the quality of the literature. Therefore, it is recommended that randomized, double-blind, placebo or controlled trials should be conducted to provide additional evidence. Second, this literature base has substantial heterogeneity. Different measures of diagnosing seizures and different drug doses of PB all lead to heterogeneity. Finally, although we used clinical or electrical seizures as the outcome index in accordance with previous studies, subgroup analysis showed that different measures of diagnosing seizures led to heterogeneity. Considering the atypical manifestations of neonatal seizures, it is suggested that EEG monitoring should be used to diagnose neonatal seizures to evaluate the severity of the convulsions, the time needed to control the seizures, and the efficacy of the drugs.

### Strengths

Our results were consistent with another study (45). That study evaluated the efficacy of LEV vs. PB and found that there was low quality evidence suggesting that LEV might not be more effective than PB. At the same time, that study did not analyze the efficacy of LEV and PB by different diagnostic methods of seizures (clinical seizures or electrical seizures) and did not compare the BSID scores between LEV and PB. In our meta-analysis, we adopted strict inclusion criteria and included high-quality literature and performed subgroup analyses for different diagnostic methods of seizures and different side effects, which supports the reliability of the results of this study. Our sensitivity analyses confirmed the stability of the results.

### Conclusion

PB is a first-line AED drug recommended by the WHO for the treatment of neonatal seizures. The new AEDs LEV may not have better efficacy than PB. At the same time, LEV is associated with a better neurodevelopment outcome and a lower risk of adverse effects. In addition, continuous EEG monitoring should be used to diagnose neonatal seizures to evaluate the severity of seizures, remission, and drug efficacy.

## Data Availability Statement

The original contributions presented in the study are included in the article/Supplementary Material, further inquiries can be directed to the corresponding author/s.

## Author Contributions

M-YQ and H-TC conceptualized and designed the study, analyzed the data, and drafted and revised the manuscript. M-YQ and L-ZZ designed the data collection instruments, collected data, and carried out the initial analyses. Q-XC and J-kM conceptualized and designed the study, coordinated, and supervised data collection, and critically reviewed the manuscript for important intellectual content. Q-XC: contributed greatly to design, correction, and supplement of the study. All authors approved the final manuscript as submitted and agreed to be accountable for all aspects of the work.

## Funding

This study was supported by Key Projects of Chongqing Health Commission: 2019ZY013201; High-level Medical Reserved Personnel Training Project of Chongqing; Chongqing Science and Technology Commission Social Livelihood Science and Technology Project: cstc2017shmsA130001; Key Projects of Chongqing Health Commission: 2017ZDXM029; and Chongqing Science and Technology Bureau (cstc2019jscx-msxmX0249).

## Conflict of Interest

The authors declare that the research was conducted in the absence of any commercial or financial relationships that could be construed as a potential conflict of interest.

## Publisher's Note

All claims expressed in this article are solely those of the authors and do not necessarily represent those of their affiliated organizations, or those of the publisher, the editors and the reviewers. Any product that may be evaluated in this article, or claim that may be made by its manufacturer, is not guaranteed or endorsed by the publisher.

## Abbreviations

LEV: Levetiracetam
PB: Phenobarbital
AEDs: Antiepileptic drugs
BZDs: Benzodiazepines
c-EEG: Continuous electroencephalography
a-EEG: Amplitude-integrated electroencephalography
v-EEG: Video electroencephalography
RCT: Randomized controlled trial
OR: Odds ratio
BSID: Bayley Scales of Infant development
NOS: Newcastle-Ottawa Scale.
