# Supplementary material for: Efficacy and Safety of Levetiracetam vs. Phenobarbital for Neonatal Seizures: A Systematic Review and Meta-Analysis
Source: Front Neurol. 2021 Nov 18;12:747745. doi: 10.3389/fneur.2021.747745 (PMC8636327; doi:10.3389/fneur.2021.747745)
Supplement: Supplementary file 1 [file Data_Sheet_1.docx]

**Efficacy and Safety of Levetiracetam Versus phenobarbital for Neonatal Seizure: A Systematic Review and Meta-Analysis**

Meng-Yuan Qiao^1,2,3,4^, Hong-Tao Cui^1,5^, Ling-Zhi Zhao^5^, Qi-Xiong Chen ^1,5^, Jing-kun Miao^6^

^1^ Children’s Hospital of Chongqing Medical University, Chongqing, China;

^2^China International Science and Technology Cooperation base of Child Development and Critical Disorders, Chongqing, China;

^3^Ministry of Education Key Laboratory of Child Development and Disorders, Chongqing, China;

^4^Chongqing Key Laboratory of Pediatrics, Chongqing, China;

^5^Chongqing Traditional Chinese Medicine Hospital, Chongqing, China;

^6^ Chongqing Health Center for Women and Children, Chongqing, China;

^*^Corresponding author:

Jing-kun Miao, Newborn screening center, Chongqing Health Center for Women and Children, 64 Jintang Street, Yuzhong District, Chongqing, 400014, China.

E-mail: jennamiao@aliyun.com; Telephone: 13110207118.

**Search Strategy in Databases (July 2021)**

**Table e-1. Full Literature Search on MEDLINE (Through Ovid)**

| **Search**  **Number** | **Search Description** |
| --- | --- |
| #1 | "Infant, Newborn"[Mesh] OR neonat* [Title/Abstract] OR newborn*[Title/Abstract] |
| #2 | “seizures"[Mesh] OR “epilepsy"[Mesh]” OR seizure*[Title/Abstract] OR epileps*[Title/Abstract] OR convulsi*[Title/Abstract] |
| #3 | “anticonvulsants” [Mesh] OR anticonvuls*[Title/Abstract] OR antiepileptic*[Title/Abstract] |
| #4 | phenobarbital OR levetiracetam |
| #5 | #3 OR #4 |
| #6 | #1 AND #2 AND #5 4088 |

**Table e-2. Full Literature Search on Cochrane Central Register of Controlled Trials**

| **Search**  **Number** | **Search Description** |
| --- | --- |
| #1 | Mesh descriptor: [Infant, Newborn] explode all trees |
| #2 | neonat*:ti,ab,kw or newborn*:ti,ab,kw |
| #3 | #1 or #2 |
| #4 | Mesh descriptor: [Seizures] explode all trees |
| #5 | Mesh descriptor: [Epilepsy] explode all trees |
| #6 | seizure*:ti,ab,kw or epileps*:ti,ab,kw or convulsi*:ti,ab,kw |
| #7 | #4 or #5 or #6 |
| #8 | MeSH descriptor: [Anticonvulsants] explode all trees |
| #9 | anticonvuls*:ti,ab,kw or antiepileptic*:ti,ab,kw or phenobarbital:ti,ab,kw or levetiracetam:ti,ab,kw |
| #10 | #8 or #9 |
| #11 | #3 AND #7 AND #10 198 |

**Table e-3. Full Literature Search on Web of Science**

| **Search**  **Number** | **Search Description** |
| --- | --- |
| #1 | TS=(Infant, Newborn OR neonat* OR newborn*) |
| #2 | TS=( Seizures OR Epilepsy OR seizure* OR epileps* OR convulsi*) |
| #3 | TS=( Anticonvulsants OR anticonvuls* OR antiepileptic* OR phenobarbital OR levetiracetam) |
| #4 | #1 AND #2 AND #3 1394 |

**Table e-4. Full Literature Search on EMBASE (Through Ovid)**

| **Search**  **Number** | **Search Description** |
| --- | --- |
| #1 | 'newborn'/exp |
| #2 | 'neonat*':ti,ab |
| #3 | 'newborn*':ti,ab |
| #4 | #1 OR #2 OR #3 |
| #5 | 'seizure'/exp OR 'epilepsy'/exp |
| #6 | 'seizure*':ti,ab OR 'epileps*':ti,ab OR 'convulsi*':ti,ab |
| #7 | #5 OR #6 |
| #8 | 'anticonvulsive agent'/exp |
| #9 | 'anticonvuls*':ti,ab OR 'antiepileptic*':ti,ab OR 'phenobarbital':ti,ab OR 'levetiracetam':ti,ab |
| #10 | #8 OR #9 |
| #11 | #4 AND #7 AND #10 6507 |

**Table e-5. Full Literature Search on Clinicaltrail.gov**

| **Search**  **Number** | **Search Description** |
| --- | --- |
| #1 | condition or disease: Neonatal Seizure |
| #2 | intervention/treatment: Anti-Epileptic |
| #3 | #1 AND #2 17 |

**Table e-6. Full Literature Search on China national knowledge internet (CNKI)**

| **Search**  **Number** | **Search Description** |
| --- | --- |
| #1 | Mesh: infant, newborn |
| #2 | Mesh: seizure |
| #3 | Mesh: epilepsy |
| #4 | #2 OR #3 |
| #5 | Mesh: anticonvulsants |
| #6 | Mesh: phenobarbital |
| #7 | Mesh: levetiracetam |
| #8 | #5 OR #6 OR #7 |
| #9 | #1 OR #4 OR #8 230 |
